# Perception of healthcare professionals about the knowledge of people living with HIV regarding clinical trials for HIV-related cancers

**DOI:** 10.1186/s12889-025-23137-w

**Published:** 2025-07-03

**Authors:** Jessica Hernández-Marrero, Tanialy Rivera-Santiago, Mariela Bournigal-Feliciano, Vivian Colón-López, Ana I. Velázquez, Jorge Salmerón, Joel M. Palefsky, Ana P. Ortiz, Marievelisse Soto-Salgado

**Affiliations:** 1 Division of Cancer Control and Population Sciences, University of Puerto Rico Comprehensive Cancer Center, San Juan, Puerto Rico, USA; 2 School of Medicine, Division of Hematology/Oncology, University of California San Francisco, San Francisco, CA USA; 3 UCSF Helen Diller Family Comprehensive Cancer Center, San Francisco, CA USA; 4 National Institute of Public Health, Epidemiology, Cuernavaca-Morelos, México and Center for Policy, Population and Health Research, National University, Mexico City, USA; 5 School of Medicine, Department of Medicine, University of California San Francisco, San Francisco, CA USA; 6 Department of Biostatistics and Epidemiology, University of Puerto Rico Medical Sciences Campus, Graduate School of Public Health, San Juan, Puerto Rico, USA; 7 First Floor, Division of Cancer Control and Population Sciences, University of Puerto Rico Comprehensive Cancer Center, PMB 371, PO Box 70344, San Juan, Puerto Rico, 00936 USA

**Keywords:** Clinical trials, Perception, Healthcare providers, HIV-related cancers

## Abstract

**Background:**

Advances in HIV treatments have prolonged the lifespan of people living with HIV (PLWH), thereby increasing the risk of chronic conditions such as HIV-related cancers. Clinical trials (CT) play an important role in advancing cancer care in PLWH. However, their underrepresentation in CT underscores the need for involvement of healthcare professionals in CT referrals. This study aims to describe healthcare professionals’ perception of PLWH understanding of CT for HIV-related cancers.

**Methods:**

We analyzed survey data from a training activity provided to healthcare professionals (*n* = 82) from HIV-specialized clinics and other healthcare organizations in Puerto Rico to assess healthcare professionals’ perception of PLWH knowledge regarding CT for HIV-related cancers.

**Results:**

Most participants were allied healthcare professionals (39.0%) or social services professionals (43.9%), with 70.8% having 1–15 years of experience caring for PLWH. Most participants believed that PLWH had limited knowledge about HIV-related cancers (65.8%) and CT for these cancers (59.8%). Nearly a quarter (23.1%) of healthcare professionals reported hearing expressions of fear regarding CT from PLWH. Conversely, almost half (49.4%) heard positive comments or experiences about participating in CT. The training activity did not include physicians. Perceived barriers for participation of PLWH in CT, according to the healthcare professionals, included fear of the unknown (48.8%), stigma associated with the participation in HIV-related clinical trials (43.9%), and lack of knowledge (42.7%).

**Conclusions:**

According to healthcare professionals, the participation of PLWH in HIV-related clinical trials could be affected by limited knowledge or awareness about their benefits. Training activities are needed to increase awareness, knowledge and improve the participation of PLWH in HIV-related CT.

**Clinical trial number:**

Not applicable.

**Supplementary Information:**

The online version contains supplementary material available at 10.1186/s12889-025-23137-w.

## Background

Advances in HIV treatments have prolonged the lifespan of people living with HIV (PLWH) [1], thereby increasing their risk of developing chronic conditions such as HIV-related cancers [2]. These related cancers are categorized into two groups [1], AIDS-defining cancers, including non-Hodgkin lymphoma, Kaposi’s sarcoma, and cervical cancer, and [2] non-AIDS-defining cancers, such as lung, anal, Hodgkin lymphoma, oropharyngeal, liver, vulvar and penile cancer [2]. The incidence of these malignancies is higher in PLWH compared to HIV-negative individuals, particularly in Latin America and the Caribbean, where risk factors of developing cancers such as anal, penile and cervical are more prevalent [3–5]. Several factors contribute to the increased cancer incidence in PLWH, including HIV-induced immune dysfunction, impairment of cells infected by the virus, co-infection with other viruses (e.g., HBV and HCV), smoking and aging. Additionally, PLWH face an elevated risk of incidental cancers such as colorectal, breast, and prostate cancer [2].

Despite accrual challenges, clinical trials (CTs) have contributed with scientific evidence improving the quality of life and survival and developing novel treatments for cancer and HIV [6]. However, the participation of Hispanics living with HIV in CTs overall remains limited. Only 1% of Hispanics of the general population participate in CTs in the US [7]. This is also observed in trials for HIV-related cancer CTs among PLWH [8]. The underrepresentation of PLWH in CTs underscores how important it is that healthcare professionals refer this population to CTs [9, 10] and their influence in the patient’s awareness and perception of CTs. Moreover, the perception of healthcare providers about the knowledge of PLWH of CTs for HIV-related cancers can provide valuable insight into their awareness, recognizing that CTs plays an important role in advancing cancer care in PLWH. For example, the Project ACCRUAL highlights the importance of disseminating information about HIV-related cancers and CTs to increase organizational and community awareness [12]. Furthermore, the trust-based relationships between healthcare providers and PLWH may be a key factor in improving CTs participation. This study aims to describe the perception of healthcare professionals regarding PLWH’s understanding and participation in CTs for HIV-related cancers. Gaining insight into PLWH’s knowledge about CTs will allow researchers and healthcare providers to deliver accurate information, address misconceptions, and overcome potential barriers to their participation [11].

## Methods

We analyzed survey data from a training activity for healthcare providers from non-profit, community-based and HIV specialized clinics in Puerto Rico (PR) held in January 2022. This activity aimed to provide capacity building for healthcare professionals in CTs, HPV-related cancer risk among PLWH, and patient navigation services and roles. The activity was designed to case managers, social workers, professional counselors, health educators, lay health workers, and nurses who routinely coordinated clinical care for PLWH and were familiar with their needs.

The survey was an adaptation and Spanish translation from the Project Accrue survey [12]. In this activity, 82 healthcare professionals completed the survey, consisting of 13 questions that explored their perception about the knowledge, participation, and barriers and facilitators for HIV-related cancer CTs among PLWH. Descriptive statistics were used to analyze the data using STATA, a statistical software for data visualization and analysis.

## Results

### Demographic information

The demographic information of study participants is presented in Table 1. The mean age of the participants was 45 ± 12.2 years, and the majority were female (72.0%). Most respondents worked at community-based organizations (79.3%), followed by the Immunology Clinics of the Puerto Rico Department of Health (11.0%) and research or academic institutions (6.1%). Participant’s roles included case manager, care technician or patient navigator (36.6%), health educator (14.6%), clinical coordinator or supervisor (12.2%), psychologist or counselor (11.0%), social worker (9.8%); nurse (8.5%), health promoter or community outreach (6.1%) and epidemiology technician (1.2%). The training activity did not include physicians, as it focused on healthcare professionals who provide non-medical, day-to-day patient care. The mean time caring for PLWH was 11.0 ± 14.6 years and the majority reported that they had not previously received training about CTs before (79.3%).

**Table 1:** Sociodemographic characteristics of the study population (*n* = 82)

Characteristic	*n*	(%)
**Age (years)**		
Mean (± SD)	45.0 (± 12.2)	--
**Sex at birth**		
Male	20	25.3
Female	59	74.7
**Role of the participant in their clinic**		
Allied-Healthcare*	32	39.0
Social Services	36	43.9
Other	14	17.1
**Profession**		
Nurse	14	17.1
Psychologist	8	9.8
Social worker	13	15.9
Professional counselor	6	7.3
Health educator	14	17.1
Other	27	33.0
**Mean (± SD) of time caring for PLWH**	11.0 ± 14.6	--
**PLWH currently participating in CTs** **(Best knowledge of healthcare providers)**		
None	20	25.0
1–4	12	15.0
5–10 people	8	9.9
>10 people	11	13.6
Unknown	30	37.0

*Physicians are not represented

### Perception of knowledge about HIV-related cancers CTs

**Fig. 1:**
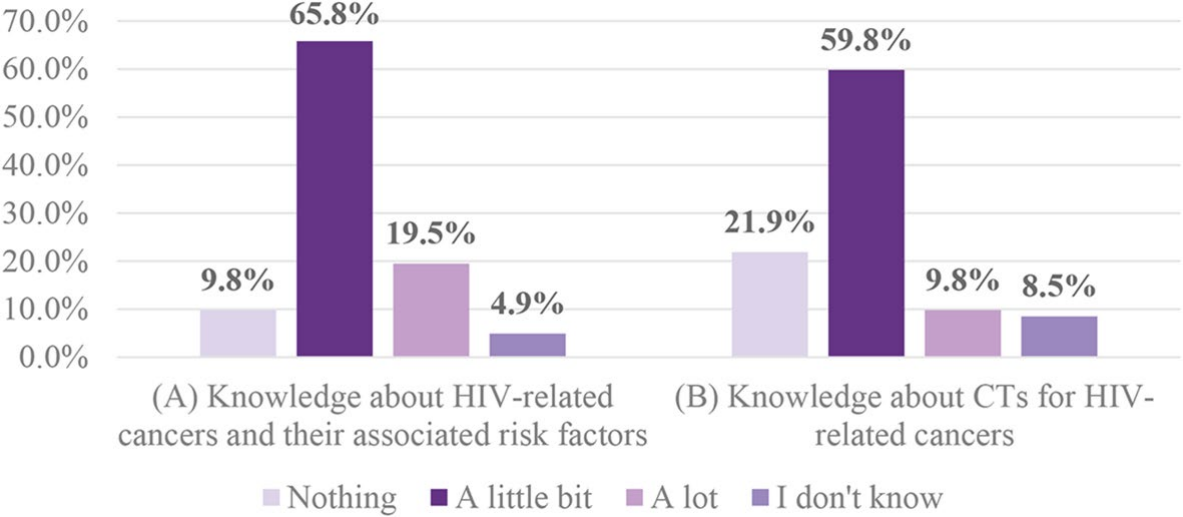
Healthcare providers’ perception of PLWH knowledge about (**A**) HIV-related cancers and their risks and (**B**) HIV-related cancer CTs (*n* = 82)

More than half of the healthcare professionals indicated that, to their knowledge, the PLWH of their community knew very little about HIV-related cancers and their associated risk factors (65.8%), or about CTs focused on HIV-related cancers (59.8%) (Fig. 1). When we asked about CTs participation, 37.0% of the respondents indicated that they did not know if the individuals to whom provide services had participated in CTs in the last 2 years, 24.7% indicated that none had participated, and 14.8% indicated that only 1–4 people had participated in CTs (Data not shown).

**Fig. 2:**
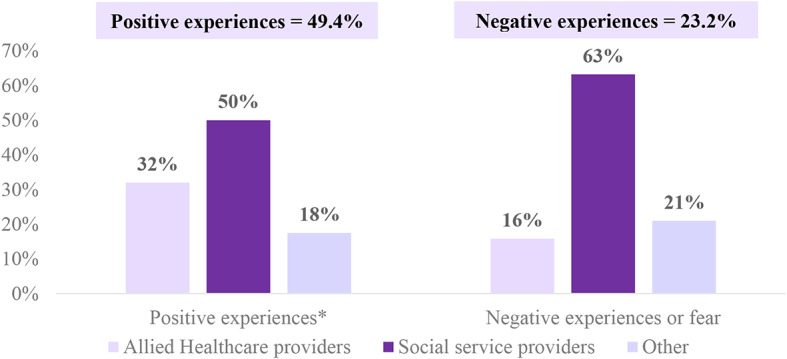
Healthcare providers’ perception of PWH experiences with CTs (*n* = 82). *1 missing

More than half (59.8%) reported that they had never heard anyone share or express fears about CTs (Data not shown). Moreover, 49.4% of the participants reported that they have heard people sharing positive comments or experiences about participating in CTs (Fig. 2). The healthcare providers’ perceived facilitators and barriers for PLWH to join CTs for HIV-related cancers is presented in Fig. 2. Most participants indicated that the principal facilitators for participating in CTs include knowing that PLWH have a greater risk of developing HIV-related cancers (21.4%), receiving free treatment or incentive for their time (18.2%), that the CTs are conducted in an organization that provides services to PLWH (13.6%), received a recommendation by a doctor (10.3%), and want better cancer treatment (9.9%) (Table 2). The principal barriers to participating in CTs, according to the healthcare providers, include are lack of knowledge about CTs benefits or CTs in general (21.4%), fear of the unknown (18.2%), stigma related to being in a CT for HIV-related cancers (13.6%), distance from home to the trial center or lack of transportation (10.3%) and feeling like guinea pigs (9.9%) (Table 2).

**Table 2:** Healthcare providers’ perceived *facilitators* and *barriers* for PLWH to join CTs for HIV-related cancers

Facilitators	%	Barriers	%
Knowing that PLWH have a greater risk of developing HIV-related cancers	21.4%	Lack of knowledge about CTs benefits or CTs in general	21.4%
Receiving free treatment or incentive for their time	18.2%	Fear of unknown	18.2%
That the CTs are conducted in an organization that provides services to PLWH	13.6%	Stigma related to being in a trial for HIV-related cancers	13.6%
Recommended by doctor	10.3%	Distance from home to trial center or lack of transportation	10.3%
Want better cancer treatment	9.9%	Feeling like guinea pigs	9.9%
Referral from other person who participate in CTs	4.1%	Thinking they are not eligible	4.1%
Other	22.6%	Other	22.6%

The participants were asked about what partnerships they thought could help raise awareness and encourage participation on HIV-related cancers CTs. Most participants answered that these include hearing information from the doctors and scientists who run the trials (93.9%), that a health professional gives them information about the topic (88.9%), hearing information about CTs from someone who has participated in a trial (78.1%), having written information such as brochures (73.4%), and having written information they can take home to discuss with friends and family (64.6%) (Data not shown).

The majority of the healthcare providers who answered the survey didn’t know where to find information about CTs of HIV-related cancers for their patients (64.6%) or information about CTs of HIV-related cancers that could benefit their patients (63.0%). When we asked about what information, in their experience, their patients will need to help them consider joining a CTs for HIV-related cancers, the three most commonly reported suggestions included information about HIV-related cancers (73.2%), benefits of the CT (directly to the patient) (70.7%), and safety information about CTs (65.8%). Lastly, they reported that the principal needs of their organization to create partnerships to raise awareness about HIV-related cancers and CTs include offering training to staff (74.4%), providing informational materials such as pamphlets (61.0%), and educational sessions for patients (59.8%) (Data not shown).

## Discussion

Clinical knowledge about HIV, cancer, as well as effective and secure treatments for PLWH has primarily resulted from research efforts. These advances have contributed to an aging population facing other chronic conditions like cancer. Thus, the participation of underrepresented minorities and PLWH in CTs is crucial, requiring their engagement and the support of their healthcare providers. The objective of this study was to describe the perception of healthcare professionals regarding PLWH’s understanding and participation of CTs for HIV-related cancers. The results can highlight the barriers for this population to participate in CTs based on the perception of the healthcare professionals.

According to healthcare professionals, the involvement of PLWH in HIV-related CTs can be affected by limited knowledge or awareness about the benefits of their participation in CTs. Similar to the Project Accrue [12], the majority of PLWH, in the opinion of healthcare providers, knew little or nothing about HIV-related cancers and their risk factors, or CTs for HIV-related cancers. This lack of knowledge about CTs could be challenging for cancer-related CTs accrual [8, 12]. Thus it is important to explore the facilitators and barriers to participation in CTs.

The facilitators for PLWH to join HIV-related cancer CTs in PR differed from those reported by Project ACCRUE, where the informants were members of the community advisory board and directors of organizations serving PLWH [12]. In PR, the main facilitators reported by the healthcare professionals were, knowing the risk of HIV-related cancers, CTs conducted at organizations serving PLWH, and that they would receive free treatment. In Project ACCRUE [12], the main facilitators for PLWH to join HIV-related cancer CT were safety information about CTs, information specific to cancer among PLWH/AIDS, and the benefits of the trial. A high level of trust in healthcare professionals was important for strong participation in CTs according to previous studies performed in PR with PLWH and women by Ortiz et al., [13] and Rivera-Díaz et al., [14] respectively. To increase awareness and determining participation in CTs among PLWH thrusting relationships with scientists, and healthcare professionals [15], hearing from individuals who have participated in CTs or written information where important. Whereas in the ACCRUE study [12], hearing from someone who participates in CTs was considered extremely helpful to increase awareness about CTs. These aspects should be considered when promoting and recruiting PLWH to cancer-related CTs.

The principal barriers when considering CTs participation reported by the key informants in the ACCRUE [12] study were similar to those mentioned by participants in PR, i.e., lack of knowledge about CTs in general, fear of the unknown, and distance to the trial site. Additionally, the stigma related to being in a CT for HIV-related cancers and feeling like guinea pigs were identified as barriers for HIV-related cancer CT participation in PR, as reported by the healthcare providers. Through the years stigma and distrust negatively affected the engagement of PLWH in their clinical management and research [16, 17], and this remains a challenge for healthcare providers and researchers.

Unfortunately, most of the healthcare providers who participated in PR didn’t know where to find information about CTs of HIV-related cancers for their patients, differing from the participants from North Carolina [12] who knew that they could find this information at medical centers, hospitals or the internet. This could represent a limitation for the participation of Puerto Rican PLWH in CTs that need further evaluation. It is important to educate healthcare professionals about where they can find information about CTs for HIV-related cancers that they can provide this information to their patients. Moreover, our participants reported that the principal needs of their organizations to raise awareness about HIV-related cancers and CTs are offering training to staff and providing informative materials and educational sessions for their community.

The limitations of this study include its small sample size and the lack of generalizability to the population of healthcare providers that care for PLWH in PR due to its convenience (non-probabilistic) sampling. Nonetheless, to our knowledge, this study was the first to describe healthcare providers’ perception of PLWH about the knowledge and participation of Puerto Rican PLWH about CTs for HIV-related cancers. Given the high burden of cancer and HIV in PR and the low participation of Hispanics in cancer-related CTs including Puerto Ricans, recognizing this information is crucial for advancing care in PLWH in PR. Further research should identify targeted interventions to increase knowledge of PLWH about CTs and their participation in CTs for HIV-related cancers.

## Conclusions

Participation of PLWH in HIV-related cancers CTs is critical. Building trustful relationships with healthcare professionals plays an important role in introducing the concept of CTs and facilitating the participation of PLWH in CTs [18]. Training activities for both patients and healthcare providers are needed to increase awareness and knowledge to improve the participation of PLWH in CTs. Increasing the knowledge and awareness of cancer-related CTs among healthcare professionals can be an effective strategy to enhance the participation of PLWH and to develop trusting relationships among patients and researchers.

## Electronic supplementary material

Below is the link to the electronic supplementary material.

Supplementary Material 1

Supplementary Material 2

## Data Availability

Data is provided within the manuscript. The data used and/or analyzed during the current study are available from the corresponding author upon reasonable request.

## Abbreviations

PLWH: People Living With HIV
HIV: Human Immunodeficiency Virus
CTs: Clinical Trials
