# Supplementary material for: Perception of healthcare professionals about the knowledge of people living with HIV regarding clinical trials for HIV-related cancers
Source: BMC Public Health. 2025 Jul 3;25:2377. doi: 10.1186/s12889-025-23137-w (PMC12224814; doi:10.1186/s12889-025-23137-w)
Supplement: Supplementary file 1 — Supplementary Material 1 [file 12889_2025_23137_MOESM1_ESM.pdf]

TRAINING FOR HEALTH PROFESSIONALS ABOUT PATIENT NAVIGATION SERVICES AND CLINICAL TRIALS

Identification number: ______________

DEMOGRAPHIC SURVEY

This document includes questions about yourself and your perception of the knowledge of the community about HIV, HIV-related cancers, and clinical trials**. In the blank space provided, include your response.**

Demographic information

1. Age: ______

2. _____ Sex assigned at birth
   1) Female
   2) Male
   3) Refuse

3. _____ Type of clinic in which you work
   1) Immunology Clinic (CPTET) of the Puerto Rico Department of Health
   2) Private clinic, specify: ____________________________________
   3) Primary Health Center 330, specify: _______________________
   4) Other, specify: ____

4. _____ Type of profession
   1) Nurse
   2) Case manager
   3) Professional counselor
   4) Psychologist
   5) Health educator
   6) Other, specify: ____

5. How much time have you worked with the population of people living with HIV? ______ (months/years)

6. _____ Do you have any previous experience receiving formal training about patient navigation services and clinical trials?
   1) Yes, specify: ____
   2) No

Perception about community knowledge of HIV-related cancers and clinical trials

1. _____ How much do you think the community your organization serves knows about **HIV/AIDS-related cancers** and their associated risk factors?
   1) Nothing
   2) A little bit

3) A great deal
4) I don't know

2. _____ How much do you think the community your organization serves knows about **clinical trials for HIV/AIDS-related cancers**?
   1) Nothing
   2) A little bit
   3) A great deal
   4) I don’t know

3. _____ To the best of your knowledge, how many of the individuals you serve are either currently participating in a clinical trial or have participated in the past 2 years?
   1) None of them (0 people)
   2) A couple of them (1-4 people)
   3) Several of them (5-10 people)
   4) A lot of them (>10 people)
   5) I don’t know

4. _____ Have you heard anyone share or express fears about clinical trials, negative things about people's experiences with them, or perhaps negative rumors about what it's like to participate in one?
   1) Yes
   2) No
   3) I don’t know

5. _____ Have you heard anyone share positive experiences or say anything positive about the potential benefits of being in a clinical trial?
   1) Yes
   2) No
   3) I don’t know

6. To the best of your knowledge, what do you think are the <u>three most important reasons that may facilitate</u> people to join a clinical trial for HIV/AIDS-related cancer? **Circle your answer.**
   1) Improving cancer treatment
   2) Recommendation from their doctor
   3) The studies offer good treatment
   4) Learn more about cancer
   5) Receive free treatment
   6) Their family understands that it is the best option
   7) Economic incentives
   8) Providing transport and/or child care
   9) Other, specify: ____________________ _____________________________

7. To the best of your knowledge, what do you think are the three most important reasons that may prevent people from joining a clinical trial for HIV/AIDS-related cancer? **Circle your answer.**

1) Thinking they are not eligible
2) Lack of time
3) Simply do not want to be in a trial
4) Mistrust of the medical community
5) Fear of the unknown
6) Stigma related to being in a trial for HIV/AIDS-related cancer
7) Lack of knowledge about clinical trials in general
8) Lack of clarity regarding how trials will benefit others
9) Lack of transportation
10) Other, specify: ________________________________

8. We are trying to develop ways of spreading positive messages about clinical trials for HIV/AIDS-related cancers. How helpful do you think each of the following items would be for raising awareness and encouraging people to join clinical trials? **Put an X on the answer you consider is correct.**

**Premise**	**Not at all helpful (1)**	**Somewhat helpful (2)**	**Extremely helpful (3)**
Hearing information about clinical trials from someone who has participated in a trial			
Having written information (like brochures) that they can take home with them			
Hearing information from the doctos and scientists who run the trials			
Seeing a presentation about the topic			
Reading a blog on a website about clinical trials			
Getting the information from their infectious disease doctors			

9. _____ Do you know where to go to find information about HIV/AIDS-related cancers for your clients?

1) Yes, specify: ____
2) No

10. _____ Do you know where to go to learn about open clinical trials for HIV/AIDS-related cancers that could benefit your clients?

1) Yes, specify: ____
2) No

11. What three kinds of information do you feel your clients most need to help them consider joining a clinical trial for HIV/AIDS-related cancer? **Circle your answer.**

1) Safety information about clinical trials
2) Information about HIV/AIDS
3) Information specific to cancer among people living with HIV/AIDS
4) Ethical responsibility of the research team during clinical trials
5) Time commitments associated with clinical trials
6) Troubleshooting when things don't go as expected in a trial
7) Benefits of the trial (directly to the patient)
8) Benefits of the trial (indirectly to society)
9) Other, specify: ____

12. To engage in a partnership to help raise awareness of HIV/AIDS-related cancers and clinical trials, what kinds of resources would your organization need the most**? Circle your answer. You can choose more than one alternative.**

1) Staff training
2) Informational materials (pamphlets, "goodies", etc.) to distribute
3) Educational sessions/training for the community
4) A book of resources that you can use or to which you can refer people
5) Someone you can call to ask questions
6) A list of funding opportunities for organizations like yours
7) Other, specify: ___________________________

13. How can we (CAMPO Consortium) best partner with you and your organization to raise awareness about HIV/AIDS clinical trials and encourage people to participate? (Select all that apply). **Circle all that apply.**

1) Provide presentations about HPV-related cancers
2) Provide information (e.g., pamphlets, flyers, information through social media, etc.) that you can give to your patients to tell them about our organization and about clinical trials
3) On-site recruitment at the clinic you work in
4) Provide banners or flyers that you can hang in your clinics
5) Other, specify: ___________________________

14. In your opinion, what factors would motivate the participation of your community in clinical trials? **For each premise, put an X in the corresponding space.**

**Premise**	**Would motivate participation (1)**	**Would not motivate participation (2)**	**Would not influence their decision (3)**
A person who participated in clinical trials recommended that they participate.			
That a family member or friend recommends that they participate.			
That their doctor is part of the research team.			
That some members of their research team are part of their cultural or ethnic group.			
That the researchers are part of a well-known hospital or university.			
The government pays for the study.			
That a private institution pays for the study.			
Receiving economic compensation.			
Providing transportation, if needed.			
Provide childcare, if needed.			
Provide food or snacks.			
Conducting the study in a site that provides services for people living with HIV.			
Conducting the study in a site near their community.			
Conducting the study during weekdays (Monday through Friday)			
Conducting the study during the weekends (Saturday and Sunday)			
