# Supplementary material for: Perception of healthcare professionals about the knowledge of people living with HIV regarding clinical trials for HIV-related cancers
Source: BMC Public Health. 2025 Jul 3;25:2377. doi: 10.1186/s12889-025-23137-w (PMC12224814; doi:10.1186/s12889-025-23137-w)
Supplement: Supplementary file 2 — Supplementary Material 2 [file 12889_2025_23137_MOESM2_ESM.pdf]

ENCUESTA DEMOGRÁFICA y PERCEPCIONES SOBRE LOS ENSAYOS CLINICOS

Este documento incluye preguntas sobre usted y sobre su percepción del conocimiento de la comunidad a la que usted sirve sobre los cánceres relacionados con el VIH y los ensayos clínicos. Favor de responder todas las preguntas. **En el espacio en blanco, incluya su respuesta.**

Número de identificación: __________

A. Información demográfica

1. Edad: ______

2. _____ Sexo asignado al nacer
   1. Femenino
   2. Masculino
   3. Rehúsa

3. _____Tipo de clínica en la que usted trabaja
   1. Clínica de Inmunología (CPTET) del Departamento de Salud de Puerto Rico, especifique el municipio: ____
   2. Clínica privada, especifique: ____
   3. Centro de Salud Primaria 330, especifique: _____________________________________
   4. Otro, especifique: ____

4. _____ ¿Cuál es su profesión?
   1. Enfermería
   2. Psicología
   3. Trabajo social
   4. Consejería profesional
   5. Educación en salud
   6. Otro, especifique: ____________________

5. _____ ¿Cuál es el puesto o rol que tiene en la organización dónde trabaja actualmente?
   1. Enfermero
   2. Manejador de caso
   3. Consejero profesional
   4. Psicólogo
   5. Educador en salud
   6. Promotor de salud
   7. Navegador de pacientes
   1) Trabajador social
   2) Otro, especifique: ____

6. Tiempo trabajando con la población de personas que viven con VIH: _____ meses _____ años
   Nota: Si usted trabaja en una organización que no provee servicios a personas que viven con VIH, escriba 999 en el espacio provisto en meses.

7. _____¿Ha recibido previamente entrenamiento formal sobre servicios de navegación de pacientes?
    1. Sí, especifique: ____
    2. No

8. _____ ¿Ha recibido previamente entrenamiento formal sobre ensayos clínicos?
    1. Sí, especifique: ____
    2. No

9. _____ ¿Provee usted u otra persona en su organización servicios de navegación a sus pacientes?
    1. Sí
    2. No
    3. No sabe

10. ¿Cuáles son las actividades principales que el navegador de pacientes de su organización realiza? *Circule todas las que aplique.*
    1. No sabe
    2. Educación a la comunidad
    3. Educación a pacientes
    4. Coordinación de cuidado
    5. Consejería financiera
    6. Consejería psicológica
    7. Otro, especifique: ____

B. Percepción sobre el conocimiento de la comunidad a la que sirve sobre los cánceres relacionados con el VIH y ensayos clínicos

1. _____ ¿Cuánto cree que conoce la población a la que sirve su organización sobre los **cánceres relacionados con el VIH y los factores de riesgo asociados**?
    1. Nada
    2. Poco
    3. Mucho
    4. Desconoce

2. _____ ¿Cuánto cree que conoce la población a la que sirve su organización sobre los **ensayos clínicos de cánceres relacionados con el VIH**?
    1. Nada
    2. Poco
    3. Mucho
    4. Desconoce

3. _____ Según su conocimiento, ¿cuántos pacientes en su organización **están participando en un ensayo clínico o han participado en un ensayo clínico en los pasados 2 años**?
    1. Ninguno (0 personas)
    2. Algunos (1-4 personas)
    3. Varios (5-10 personas
    4. Muchos (>10 personas)
    5. Desconoce

4. _____ ¿Ha escuchado a alguien compartir o expresar miedo sobre los ensayos clínicos, comentarios negativos sobre las experiencias de otras personas o rumores negativos sobre participar en algún ensayo clínico?
    1. Sí
    2. No
    3. Desconoce

5. _____ ¿Ha escuchado a alguien compartir comentarios positivos o experiencias positivas sobre participar en los ensayos clínicos?
    1. Sí
    2. No
    3. Desconoce

6. En su opinión, ¿cuáles son las <u>tres razones principales que facilitan que las personas que viven con VIH participen en ensayos clínicos</u> sobre cánceres relacionados con el VIH? **Circule las tres razones principales.**
    1. Tener el conocimiento de que las personas que viven con VIH están a mayor riesgo de desarrollar cánceres relacionados con el VIH
    2. Que una persona que ya participó en un ensayo clínico le recomiende participar
    3. Querer mejorar el tratamiento para el cáncer
    4. Recomendación de su doctor
    5. Qué su doctor sea parte del grupo de investigadores del ensayo clínico
    6. Los estudios ofrecen buenos tratamientos
    7. Obtener más información sobre el cáncer
    8. Es la única opción viable de tratamiento
    9. Recibir tratamiento gratuito
    10. Su familia entiende que es la mejor opción
    11. Recibir una compensación económica por su tiempo
    12. Se provee transportación
    13. Quieren contribuir al conocimiento científico
    14. Que los ensayos clínicos se lleven a cabo en una organización que provean servicios a personas que viven con VIH
    15. Otro, especifique: ____

7. En su opinión, ¿cuáles son las tres barreras principales para que las personas que viven con VIH participen en ensayos clínicos sobre cánceres relacionados con el VIH? **Circule las tres razones principales.**
    1. Pensar que no son elegibles
    2. Falta de tiempo
    3. Trabajo
    4. Estudio
    5. No quieren participar en un ensayo clínico
    6. El doctor no se lo recomendó
    7. No confían en los investigadores
    8. Miedo a lo desconocido
    9. Estigma relacionado a participar en un ensayo clínico sobre cánceres relacionados con el VIH
    10. Falta de conocimiento sobre los ensayos clínicos en general
    11. Falta de conocimiento sobre cómo los ensayos clínicos benefician a los demás
    12. Falta de transportación
    13. Distancia entre el lugar de residencia y el lugar dónde se llevan a cabo los ensayos clínicos
    14. Le preocupa que su información personal no se maneje de manera confidencial
    15. Sentirse que son usados como conejillos de india
    16. Otro, especifique: ______________________________

8. Estamos tratando de buscar formas de promover mensajes positivos sobre los ensayos clínicos de cánceres relacionados con el VIH. ¿Cuánto cree usted que cada de las siguientes estrategias nos ayudaría a crear conciencia y a promover la participación de personas que viven con VIH en ensayos clínicos? **Coloque una X en el recuadro de la respuesta que entiende es correcta.**

**Premisa**	**Nada útil (1)**	**Algo útil (2)**	**Muy útil (3)**
Escuchar información sobre ensayos clínicos de una persona que ha participado en algún estudio.			
Tener información escrita (como folletos o volantes) que puedan llevarse a sus casas.			
Tener información escrita (como folletos o volantes) para discutir con sus familiares o amigos.			
Escuchar información de los doctores y científicos que trabajan en el ensayo clínico.			
Que un profesional de la salud le brinde información sobre el tema.			
Leer información en internet (por ejemplo, en un blog) sobre ensayos clínicos.			
Obtener la información sobre ensayos clínicos por parte de médicos especialistas en enfermedades infecciosas (infectólogos).			

9. _____ ¿Sabe dónde conseguir información sobre ensayos clínicos de cánceres relacionados con el VIH para sus pacientes?
    1. Sí, especifique: ____
    2. No

10. _____ ¿Sabe dónde puede aprender sobre ensayos clínicos de cánceres relacionados con el VIH que puedan beneficiar a sus pacientes?
    1. Sí, especifique: ____
    2. No

11. Seleccione <u>tres tipos de información</u> que usted entiende que su comunidad necesita saber para ayudarlos a considerar su participación en algún ensayo clínico. **Circule tres alternativas.**
    1. Información sobre seguridad en ensayos clínicos
    2. Información sobre el VIH/SIDA
    3. Información sobre cánceres relacionados con el VIH
    4. Responsabilidad ética del equipo de investigación en los ensayos clínicos
    5. Compromisos de tiempo asociados a la participación en los ensayos clínicos
    6. Solución de problemas cuando se obtiene un resultado no esperado en un ensayo clínico
    7. Beneficios del ensayo clínico (directamente al paciente)
    8. Beneficios del ensayo clínico (indirectamente a la sociedad)
    9. Otro, especifique: ____

12. ¿Qué tipos de recursos entiende usted que su organización necesitaría para crear alianzas con el fin de crear conciencia sobre los cánceres relacionados con el VIH y los ensayos clínicos? **Circule su respuesta. Puede marcar más de una alternativa.**
    1. Ofrecer entrenamiento a los empleados
    2. Proveer material educativo para distribuir (e.g., folletos informativos)
    3. Ofrecer sesiones educativas a la comunidad
    4. Un libro de recursos con información sobre ensayos clínicos y cánceres relacionados con el VIH, para utilizar con los pacientes o para referir a los pacientes
    5. Identificar una persona a la que se pueda llamar para realizar preguntas
    6. Proveer una lista de oportunidades de financiamiento para organizaciones cómo la suya
    7. Otro, especifique: ____

13. ¿Cómo el estudio CAMPO podría crear una alianza con usted y su organización para crear conciencia sobre los ensayos clínicos de cánceres asociados al virus del papiloma humano (VPH) en personas que viven con VIH y animar a las personas a participar? **Circule todas las opciones que apliquen.**
    1. Proveer presentaciones o charlas sobre cánceres asociados al VPH
    2. Proveer información (e.g., folletos informativos, volantes/flyers) que se le pueda compartir a los pacientes para que aprendan sobre nuestra organización y los ensayos clínicos que se están llevando a cabo en nuestra organización
    3. Ir presencialmente a reclutar participantes en sus clínicas
    4. Proporcionar banners o flyers de nuestros estudios para que puedan colocarlos en su clínica
    5. Otro, especifique: ____
